# Supplementary material for: Uptake of Skilled Maternal Healthcare in Ethiopia: A Positive Deviance Approach
Source: Int J Environ Res Public Health. 2020 Mar 5;17(5):1712. doi: 10.3390/ijerph17051712 (PMC7084325; doi:10.3390/ijerph17051712)
Supplement: Supplementary file 1 [file ijerph-17-01712-s001.zip › Suupp file/Additional figure 1.pdf]

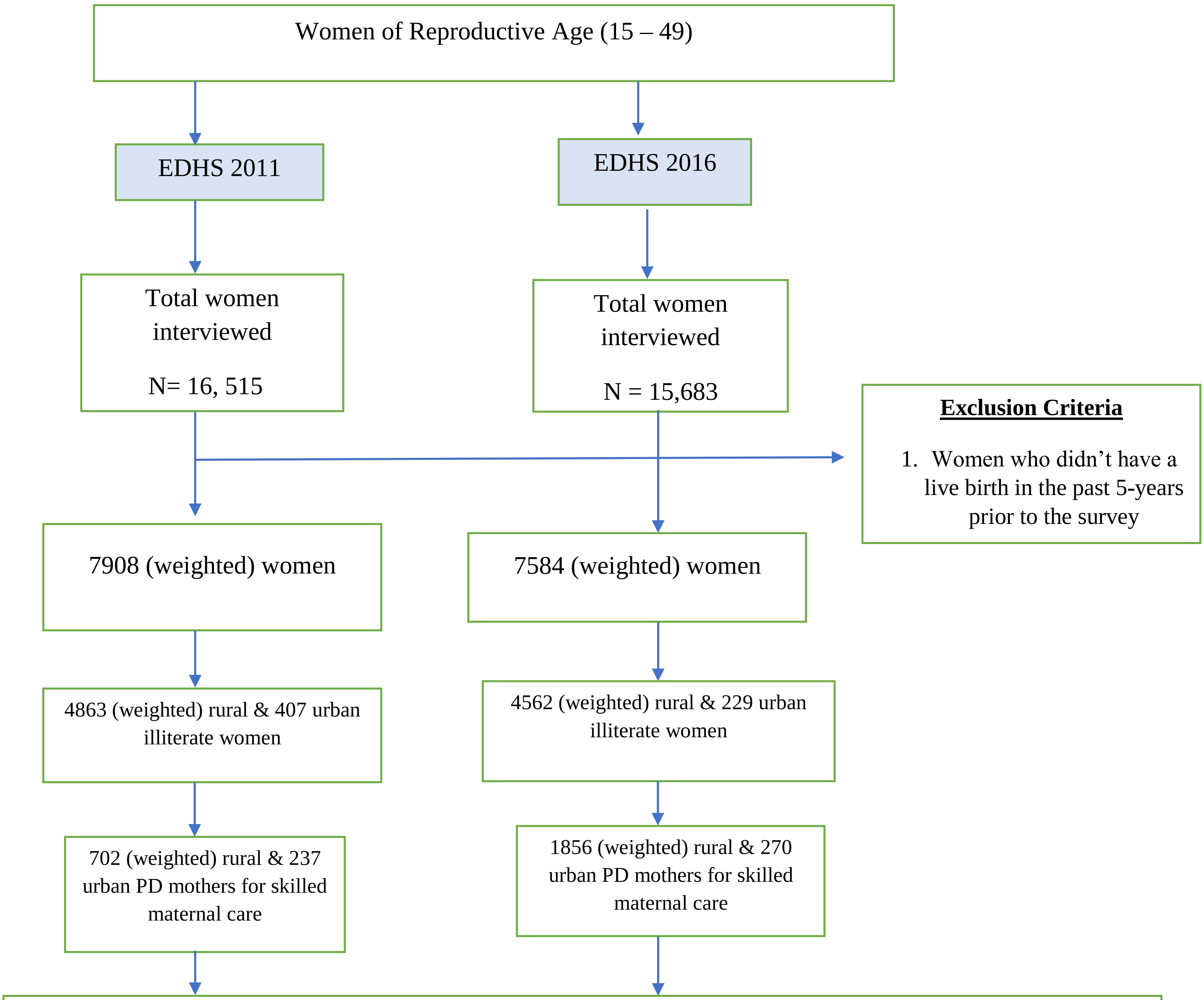

Fig S1. Schematic presentation showing sampling in the 2011 and 2016 Ethiopian Demographic Health Surveys for identification of Positive Deviants on skilled maternal healthcare use
